# Discovering Antioxidant Molecules in the Archaea Domain: Peroxiredoxin Bcp1 from* Sulfolobus solfataricus* Protects H9c2 Cardiomyoblasts from Oxidative Stress

**DOI:** 10.1155/2016/7424870

**Published:** 2016-09-26

**Authors:** Carmen Sarcinelli, Gabriella Fiorentino, Elio Pizzo, Simonetta Bartolucci, Danila Limauro

**Affiliations:** Dipartimento di Biologia, Università di Napoli “Federico II”, Via Cinthia, 80126 Naples, Italy

## Abstract

Peroxiredoxins (Prxs) are ubiquitous thiol peroxidases that are involved in the reduction of peroxides. It has been reported that prokaryotic Prxs generally show greater structural robustness than their eukaryotic counterparts, making them less prone to inactivation by overoxidation. This difference has inspired the search for new antioxidants from prokaryotic sources that can be used as possible therapeutic biodrugs. Bacterioferritin comigratory proteins (Bcps) of the hyperthermophilic archaeon* Sulfolobus solfataricus* that belong to the Prx family have recently been characterized. One of these proteins, Bcp1, was chosen to determine its antioxidant effects in H9c2 rat cardiomyoblast cells. Bcp1 activity was measured* in vitro* under physiological temperature and pH conditions that are typical of mammalian cells; the yeast thioredoxin reductase (*y*TrxR)/thioredoxin (*y*Trx) reducing system was used to evaluate enzyme activity. A TAT-Bcp1 fusion protein was constructed to allow its internalization and verify the effect of Bcp1 on H9c2 rat cardiomyoblasts subjected to oxidative stress. The results reveal that TAT-Bcp1 is not cytotoxic and inhibits H_2_O_2_-induced apoptosis in H9c2 cells by reducing the H_2_O_2_ content inside these cells.

## 1. Introduction

Reactive oxygen species (ROS), notably, superoxide (O_2_^•−^), the hydroxyl radical (HO^•^), and hydrogen peroxide (H_2_O_2_), are potent oxidants that are generated during aerobic metabolism and in response to external factors. At high concentrations, ROS can damage all major classes of biological macromolecules, leading to protein oxidation, lipid peroxidation, DNA base modifications, and strand breaks. In addition, H_2_O_2_ plays a key role in cellular metabolism because it functions as a signalling molecule that regulates cell growth, cell adhesion, cell differentiation, and apoptosis. For these reasons, the ROS concentrations must be strictly controlled. However, the regulation of the ROS concentrations within cells is not clearly understood [1].

Living organisms have evolved different antioxidant defence systems to protect themselves against ROS toxicity. Among the antioxidant enzymes, superoxide dismutase (SOD) is involved in the dismutation reaction of O_2_^•−^ in H_2_O_2_ that in turn is converted to H_2_O by an array of enzymes, such as catalase, glutathione peroxidase (GPx), and peroxiredoxin (Prx). Recently, attention has been given to Prxs, which are ubiquitous thiol peroxidases in archaea and eukaryotes, including humans. All Prxs share a common structure characterized by the thioredoxin (Trx) fold [2]. Prxs are typically classified as either 1-Cys Prxs or 2-Cys Prxs, depending upon the cysteine residues involved in catalysis. In 2-Cys Prxs, the first cysteine is located at the N-terminus and it is known as peroxidatic cysteine (C_P_), whereas the second cysteine is called the resolving cysteine (C_R_). The C_R_ is situated at the C-terminus or in different central positions downstream of C_P_ [3, 4]. H_2_O_2_ oxidizes C_P_ to sulfenic acid (C_P_-SOH), which in turn condenses with C_R_ to form a disulfide bond. A disulfide reducing system, generally composed of thioredoxin reductase (TrxR)/thioredoxin (Trx), is coupled to Prx for recycling [5]. In some Prxs, called sensitive Prxs, H_2_O_2_ can further oxidize C_P_-SOH to its inactive forms, the sulfinic (C_P_-SO_2_H) or sulfonic (C_P_-SO_3_H) acids, thereby preventing disulfide bond formation and inactivating the enzyme. The reason for this sensitivity is due to specific structural motifs. Specifically, a “GGLG” sequence and a YF C-terminal extension stiffen and stabilize the fully folded (FF) active site, making the enzyme more susceptible to overoxidation [6–8]. For this reason, eukaryotic Prxs are termed* sensitive* in contrast to the large majority of prokaryotic Prxs, which are considered robust because they lack these motifs [9]. This structural feature acquired during the evolution endows sensitive Prxs with additional functionality beyond basic antioxidant activity, including the ability to regulate peroxide signalling in eukaryotic cells [10].

An array of bacterioferritin comigratory proteins (Bcps) belonging to Prx family, named Bcp1, Bcp2, Bcp3, and Bcp4, have been recently characterized from the hyperthermophilic archaeon* Sulfolobus solfataricus* [2, 11–13]. Archaeal Bcp1 was expressed in* E. coli* and characterized [4]. It is reported that Bcp1 regenerates through an unusual coupling mechanism. Specifically, a reduction mediated by the interaction of* Ss*TrxR (*Sso*2416) and Protein Disulfide Oxidoreductase (*Ss*PDO) (*Sso*0192) [14–16] replaces the standard Trx. Functional studies reveal that Bcp1 performs the catalytic reaction using an atypical 2-Cys mechanism, in which C_P_45 forms an intramolecular disulfide bond with C_R_50. The X-ray structure of the C45S/C50S double mutant, which represents the fully reduced enzyme state, was determined to a resolution of 2.15 Å and showed a Trx fold that was similar to other Prxs [4].

This paper shows that archaeal Prx can function as an antioxidant in eukaryotic cells. Specifically, our data show Bcp1 peroxidase activity under pH and temperature conditions that are typical of mammalian cells. Furthermore, we show that the archaeal Prx is active, although eukaryotic recycling system (different from that of* Sulfolobus*) is used [14]. A fusion protein consisting of HIV Transactivating Transduction (TAT) peptide [17] and Bcp1 was constructed to facilitate uptake into H9c2 cells. Its possible cytotoxic effects and ability to decrease the H_2_O_2_ levels inside the cells were also evaluated. Our results indicate that in an* in vitro* cellular system (i.e., H9c2 rat cardiomyoblasts), the archaeal enzyme can (1) function as an antioxidant to reduce the endogenous peroxide levels and (2) decrease cellular apoptosis following H_2_O_2_-induced stress.

## 2. Materials and Methods

### 2.1. Expression and Purification of Recombinant Proteins in* E. coli*

#### 2.1.1. Bcp1 from* S. solfataricus*

*Escherichia coli* BL21-CodonPlus (DE3)RIL/pETBcp1 [12] was grown to OD_600 nm_ of 0.8 in Luria-Bertani (LB) medium supplemented with kanamycin (10 *μ*g mL^−1^) and chloramphenicol (34 *μ*g mL^−1^) at 37°C. Bcp1 expression was induced with 1 mM isopropyl-*β*-D-thiogalactoside (IPTG) (Inalco) for 6 h at 37°C.* E. coli* cells containing the recombinant protein were harvested by centrifugation, and the pellet from a 1 L culture was suspended in 20 mL of ice-cold 20 mM Tris-HCl, pH 8.0, containing a complete EDTA-free protease inhibitor cocktail (Roche, IN, USA). The protein was purified using a previously described method [11].

#### 2.1.2. Thioredoxin (*y*Trx) and Thioredoxin Reductase (*y*TrxR) from Yeast

*E. coli* BL21-CodonPlus (DE3)RIL was transformed with pET17yTrx or pET17yTrxR [18] (both generously provided by Sang Won Kang, University of Seoul, Korea) and cultured in 1 L of LB medium supplemented with ampicillin (100 *μ*g mL^−1^) and chloramphenicol (34 *μ*g mL^−1^) at 37°C. The expression of both* y*Trx and* y*TrxR was induced with 0.5 mM IPTG for 16 h at 37°C to an OD_600 nm_ of 0.8. The* E. coli* cells were harvested by centrifugation and each pellet was suspended in 20 mL of ice-cold 20 mM Tris-HCl, pH 7.5, 10 mM NaCl, 5 mM DTT, 1 mM EDTA, and a complete EDTA-free protease inhibitor cocktail and disrupted by sonication with 5 min pulses at 20 Hz (30′′ on and 30′′ off). The soluble fractions were obtained after ultracentrifugation at 160,000 ×g for 30 min at 4°C.

The cell extract was heated at 75°C for 30 min, centrifuged at 15,000 ×g at 4°C for 30 min, and then concentrated (Amicon, Millipore Corp., Bedford, MA, USA) to purify* y*Trx. The sample was loaded on a Superdex S-75 column (30 cm × 1 cm, 25 mL) that had been equilibrated with 50 mM Tris-HCl, pH 7.5, and 0.2 M KCl and was connected to an AKTA system (GE Healthcare). The elution was performed at a flow rate of 0.4 mL min^−1^.

The sample was loaded onto a Hi-Trap DEAE FF column (7 × 25 mm, 1 mL) in 20 mM Tris-HCl, pH 7.5, and 1 mM EDTA connected to AKTA system (GE Healthcare) to purify yTrxR. The proteins were eluted with a linear gradient of 0–0.5 M NaCl in 30 min at a flow rate of 0.5 mL min^−1^.

For both proteins, the fractions were pooled, analysed by SDS-PAGE, and extensively dialysed against 20 mM Tris-HCl, pH 7.0.

### 2.2. Enzymatic Activities

#### 2.2.1. *y*Trx and* y*TrxR Activity Assays

*y*Trx activity was detected using the insulin precipitation assay [19], in a reaction mixture containing 0.1 M sodium phosphate pH 7.0, 2 mM EDTA, 1 mg mL^−1^ insulin, 1 mM DTT, and 5 *μ*M yTrx. The increase in the absorbance due to the precipitation of the insulin B chain was monitored at 650 nm in a Varian Cary 50 Bio UV-Vis spectrophotometer.

*y*TrxR activity was evaluated using the 5,5′-dithiobis(2-nitrobenzoic acid) (DTNB) reduction method [20]. The formation of the 2-nitro-5-thiobenzoate product was monitored spectrophotometrically by the increase at *A*_412 nm_ at 30°C. The reaction mixture contained 0.1 M potassium phosphate, pH 7.0, 2 mM EDTA, 0.2 mM NADPH, 0.05 mM FAD, 0.5 mM DTNB, and 0.2 *μ*M yTrxR.

#### 2.2.2. Bcp1 Activity Assays

Bcp1 peroxidase activity was tested in an assay using DTT [11]. The reaction mixture contained 0.1 M Hepes, pH 7.0, 10 mM DTT, 0.1 mM H_2_O_2_, and 10 *μ*M Bcp1. After incubation for 5 min at 37°C, the reaction was stopped adding a trichloroacetic acid solution (10% w/v). The amount of H_2_O_2_ remaining was detected by measuring the *A*_490 nm_ of the purple-coloured ferrothiocyanate complex that developed after the addition of 0.2 mL of 10 mM Fe(NH_4_)_2_(SO_4_)_2_ and 0.1 mL of 2.5 M KSCN.

The effect of pH on Bcp1 activity was analysed using 0.1 M citrate phosphate (pH 5.5–6.5) and 0.1 M Hepes (pH 7.0–7.5) buffers and the assay conditions reported above. The pH stability of Bcp1 was also evaluated after the enzyme was incubated in the buffers (pH 5.5–7.0) for 30 min, 1 h, 2 h, 4 h, and 16 h, and the residual peroxidase activity was determined under standard conditions.

The peroxidase activity was also evaluated using NADPH/yTrxR/yTrx as the electron donor system to reduce Bcp1. The assay mixture contained 50 mM Hepes, pH 7.0, 1 mM EDTA, 0.2 mM NADPH, 5–100 *μ*M yTrx, 0.1 *μ*M yTrxR, and 3.5 *μ*M Bcp1. After preincubation at 30°C for 2 min, the reaction was started by adding 0.2 mM H_2_O_2_. The NADPH oxidation was monitored spectrophotometrically at 340 nm.

### 2.3. TAT-Bcp1: Cloning, Expression, and Purification

The* bcp1* coding sequence was isolated from the pET30Bcp1 vector [12] after digestion with the* Nde*I and* Xho*I restriction endonucleases. The* bcp1* fragment was purified by agarose gel electrophoresis using the QIAquick Gel Extraction Kit (Qiagen, Milano, Italy) and cloned into the pTAT2.2 expression vector (Addgene, Cambridge, USA).* E. coli *BL21-CodonPlus (DE3)RIL competent cells were transformed with the obtained vector, pTAT2.2-Bcp1, and selected on LB agar plates with kanamycin (10 *μ*g mL^−1^) and chloramphenicol (34 *μ*g mL^−1^). The selected colony was cultured in LB medium with the same antibiotics at 37°C. The cells were grown up to an OD_600 nm_ of 0.8 and the expression of TAT-Bcp1 was induced with 1 mM IPTG for 3 h at 37°C. TAT-Bcp1 was purified using the same conditions described for Bcp1. Peroxidase activity was evaluated using the DTT assay at 37°C, as reported for Bcp1.

### 2.4. Cell Culture and Treatment

H9c2 cells (Rat Embryonic Myocardium Cells, CRL-1446) were purchased from the American Type Culture Collection (ATCC) and grown in Dulbecco's Modified Eagle Medium (DMEM) supplemented with 10% foetal bovine serum (FBS), 4 mM L-glutamine, a 1% v/v penicillin/streptomycin solution (100 U mL^−1^), and 1 mM sodium pyruvate (Euroclone, Milano, Italy) at 37°C in a 5% CO_2_ incubator. A freshly prepared H_2_O_2_ (Sigma-Aldrich, Milano, Italy) stock aqueous solution was added to the cells at a final concentration of 0.3 mM and incubated for 1 h or 3 h to induce oxidative stress.

### 2.5. Internalization of TAT-Bcp1 in H9c2 Cells

H9c2 cells were seeded in 6-well plates at a density of 2 × 10^5^ cells/well in 2 mL of medium, and then TAT-Bcp1 (0.5−10 *μ*M) was added to the cells and incubated for different times (1 h, 4 h, 16 h, and 24 h). As a control, the cells were incubated with buffer diluted in medium. The cells were then detached with trypsin-EDTA, harvested by centrifugation, and washed with PBS. The cells were lysed on ice for 20 min in 0.5% Triton X-100 in PBS containing a complete EDTA-free protease inhibitor cocktail. After centrifugation at 1,500 ×g for 10 min at 4°C, the supernatant representing the cytoplasmic fraction was collected and quantified by the Bradford method [21] using Bio-Rad Protein Assay Reagent (Bio-Rad, Germany). Fifty micrograms of cytoplasmic proteins was separated by SDS-PAGE on a gel containing 15% polyacrylamide and then examined by western blot analysis using a horseradish peroxidase- (HRP-) conjugated anti-5X His tag antibody (mAb) (Qiagen) diluted 1 : 2,000 in blocking buffer. Chemiluminescent detection was performed according to the manufacturer's recommendations (Immobilon Western Chemiluminescent HRP Substrate, Millipore, MA, USA) using the Chemidoc system (Bio-Rad). The quantitative analysis was performed using Quantity One software.

### 2.6. Cell Viability Assay

Cell viability was evaluated using the 3-(4,5-dimethylthiazol-2-yl)-2,5-diphenyltetrazolium bromide (MTT) assay [22]. H9c2 cells were seeded in 96-well plates at a density of 3.5 × 10^3^ cells/well in 0.1 mL of DMEM. After 24 h, the cells were incubated with 10 *μ*M TAT-Bcp1 for different times (1 h, 4 h, 16 h, 24 h, 48 h, and 72 h). As a control, the cells were incubated with buffer diluted in medium. The MTT solution (Sigma-Aldrich, Milano, Italy) was added at 0.5 mg mL^−1^ in DMEM without phenol red (0.1 mL/well). After a 4 h incubation at 37°C, the formazan salts were solubilized in 0.04 M HCl in anhydrous isopropanol and cell viability was evaluated by measuring the *A*_570 nm_ using an automatic plate reader spectrophotometer (VICTOR3*™* Multilabel Counter, Perkin Elmer, Shelton, CA, USA). Cell survival was expressed as the means of the percentage values compared to the control. Each sample was analysed in triplicate and the assay has been repeated at least three times.

### 2.7. Measurement of the H_2_O_2_ Content

The H_2_O_2_ levels were determined using a Fluorescent Peroxide Sensor (MAK164 Sigma-Aldrich), which generates a fluorescent product proportional to the H_2_O_2_ content following a reaction with H_2_O_2_.

The intracellular H_2_O_2_ levels were measured under (1) physiological and (2) stressed conditions.

1. H9c2 cells were pretreated or with or without increasing concentrations of TAT-Bcp1 (1–20 *μ*M) for 60 min at 37°C. Next, the cells were washed twice in PBS and lysed as reported above. Then, the samples were incubated with the Fluorescent Peroxide Sensor diluted in working solution according to the manufacturer's instructions and incubated for 30 min at 37°C. The fluorescence intensity was measured using a multimode reader synergy HT calibrated for excitation at 490 nm and emission at 525 nm.
2. H9c2 cells were pretreated with TAT-Bcp1 (10 *μ*M for 16 h) and then stressed with 0.3 mM H_2_O_2_ for 30 or 60 min at 37°C. The cells were then washed and lysed, and finally the H_2_O_2_ levels were measured using the fluorescence assay described above. Every experiment included four replicates.

### 2.8. Cell Apoptosis

H9c2 cells were seeded in 6-well plates at a density of 1.5 × 10^5^ cells/well in 2 mL of DMEM and grown to nearly 70% confluence. The cells were then incubated with 10 *μ*M TAT-Bcp1 for different times (1 h, 4 h, 16 h, and 24 h) before treatment with H_2_O_2_ (0.3 mM for 1 and 3 h).

### 2.9. EB and AO Staining Assay

Apoptotic cells were detected by ethidium bromide (EB) and Acridine Orange (AO) staining of the nuclei [23]. AO permeates all cells and stains the nuclei green, whereas EB is only incorporated in apoptotic cells, staining the nuclei red.

The cells were detached with trypsin-EDTA, washed with ice-cold PBS, resuspended in 50 *μ*L of PBS containing a 5 *μ*g mL^−1^ EB/AO dye mixture, and incubated at 37°C for 20 min. The stained cells were visualized under a fluorescence microscope (Nikon Eclipse E-100). Images were taken at 100x magnification. Both the apoptotic (red) and live (green) cells in each sample were counted in five microscopic fields.

### 2.10. PARP-1 Cleavage

Nuclear extracts were prepared by detaching the cells with trypsin-EDTA, incubating them on ice for 10 min in 10 mM Hepes, pH 8.0, containing 10 mM KCl, 1.5 mM MgCl_2_, 0.5 mM DTT, and a complete EDTA-free protease inhibitor cocktail (Roche), lysing them by adding 0.1% NP-40, and centrifuging the lysates at 1,500 ×g for 10 min at 4°C. The pellet was dissolved in RIPA buffer (50 mM Tris-HCl, pH 8.0, 150 mM NaCl, 1% NP-40, 0.5% sodium deoxycholate, and 0.1% SDS) containing a protease inhibitor cocktail and incubated on ice for 30 min. After centrifugation at 1,500 ×g for 30 min at 4°C, the nuclear protein concentration was measured using the Bradford method [21], and 30 *μ*g of the protein extracts was separated by 10% SDS-PAGE. Western blotting analysis was performed using the chemiluminescent method and the following antibodies: poly(ADP-ribose) polymerase-1 (PARP-1) mAb (C2-10, Santa Cruz, CA) diluted 1 : 2,000 in blocking buffer (5% BSA in TBS buffer containing 0.1% v/v Tween 20) or the B23 mAb (Sigma-Aldrich, St. Louis, USA; 1 : 2,000 dilution) as loading control for the nuclear proteins. An HRP-conjugated goat anti-mouse IgG was used for detection (Pierce, Rockford, IL, USA).

### 2.11. Statistical Analysis

The data for the cell viability and cell apoptosis assays are presented as the means ± SD of at least three repeats. Statistical analysis was performed using a two-tailed Student's* t*-test. A *p* value < 0.05 was defined as indicating a statistically significant difference.

## 3. Results

### 3.1. Sequence Alignment of Bcp1 with Prx Orthologues

**Figure 1:**
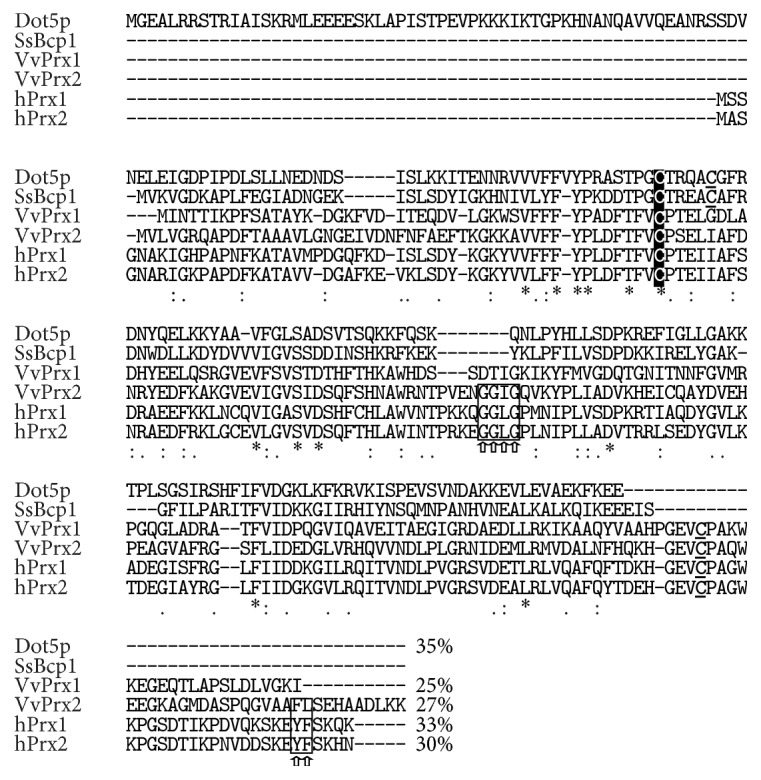
Alignment of* Ss*Bcp1 with the Prx orthologues. The multiple sequence alignment of* S. solfataricus* Bcp1 (AAK42253),* H. sapiens h*Prx1 (BAG70076) and* h*Prx2 (AAH39428),* S. cerevisiae* Dot5p (DAA08535), and* V. vulnificus Vv*Prx1 (ADV88988) and* Vv*Prx2 (ADV87477) was performed using the MUSCLE algorithm. Identical and similar amino acids are marked with asterisks and points, respectively. The peroxidatic cysteines are indicated in white and the resolving cysteines are underlined; the motifs for the sensitive Prxs are boxed and marked with arrows.

The CLUSTAL multiple alignment of Bcp1 versus human Prxs (*h*Prx1 and* h*Prx2) [24], Prx (Dot5p) from* Saccharomyces cerevisiae* [25], and prokaryotic Prxs belonging to* Vibrio vulnificus *(*Vv*Prx1 and* Vv*Prx2) [9] was performed using MUSCLE (3.8). As shown in Figure 1, Bcp1 has the highest percent identity with Dot5p and* h*PrxI of 35% and 33%, respectively. The first similarly to Bcp1 is the atypical 2-Cys Prxs, and the two enzymes are characterized by a CxxxxxC motif, in which the two cysteine residues are the C_P_ and C_R_, respectively [4, 25], but it does not retain the sensitivity motifs to overoxidation (GGLG and YT at C-terminus). The latter, which belongs to the typical 2-Cys Prxs, has a similar percent identity but has the sensitivity motif that renders it susceptible to overoxidation. On the other hand,* Vv*Prx1 and* Vv*Prx2, which belong to the prokaryotic domain, show lower identity with Bcp1 (25% and 27%, resp.), and only the first presents the overoxidation motif. This analysis suggests that prokaryotic Bcp1 could be a robust peroxiredoxin that also functions in all eukaryotic environments.

### 3.2. Bcp1 Peroxidase Activity

**Figure 2:**
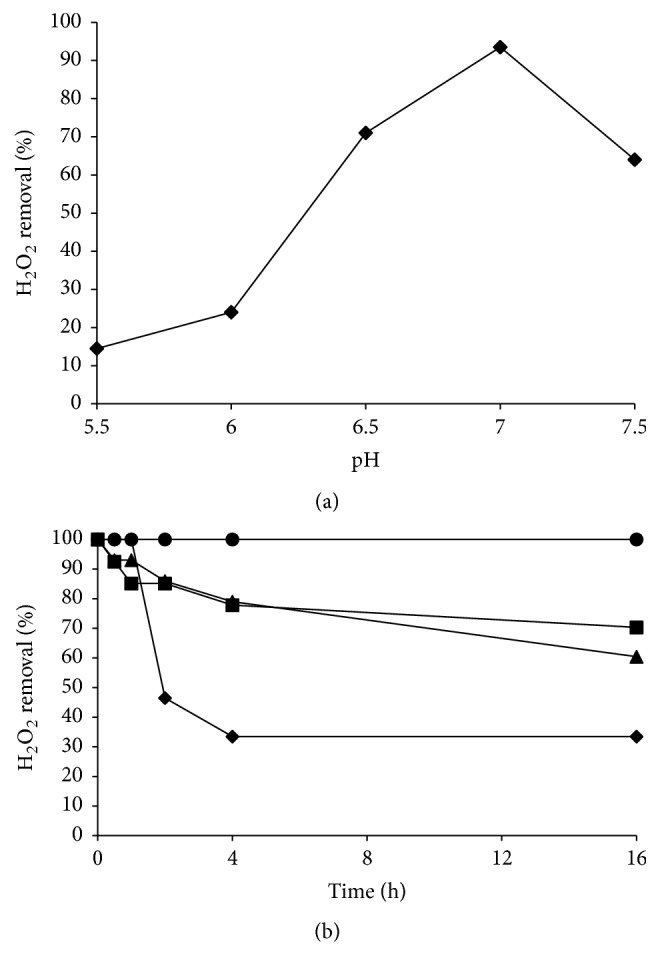
Analysis of the pH optimum and pH stability of Bcp1. H_2_O_2_ removal was measured using the ferrothiocyanate method in the presence of DTT as the electron donor at 37°C. (a) The optimum pH was measured in buffers within the range pH 5.5–7.5. (b) pH stability was determined at 37°C by incubating Bcp1 in different buffers, pH 5.5 (◆), pH 6.0 (■), pH 6.5 (▲), and pH 7.0 (●), for the following times: 30 min, 1 h, 2 h, 4 h, and 16 h.

Bcp1 was expressed in* E. coli*, purified [12], and the peroxidase activity was successively determined at 37°C and at different pH values using the DTT assay reported in the MM to detect Bcp1 peroxidase activity under the typical temperature and pH conditions of eukaryotic cells. Our results showed that Bcp1 was able to remove 100% of H_2_O_2_ at 37°C (data not shown) and that the optimal peroxidase activity was achieved at pH 7.0 (Figure 2(a)). In addition, pH stability was determined by incubating Bcp1 at different pH values (5.5–7.0) for various times and then measuring H_2_O_2_ removal (Figure 2(b)). After 16 h incubation at pH 7.0, Bcp1 retained 100% of its initial activity, while at pH 6.5, 6.0, and 5.5, the enzyme maintained 60%, 70%, and 33% of its initial activity, respectively. At pH 5.5, the relative Bcp1 activity decreased to 47% of the starting value after two hours of incubation.

**Figure 3:**
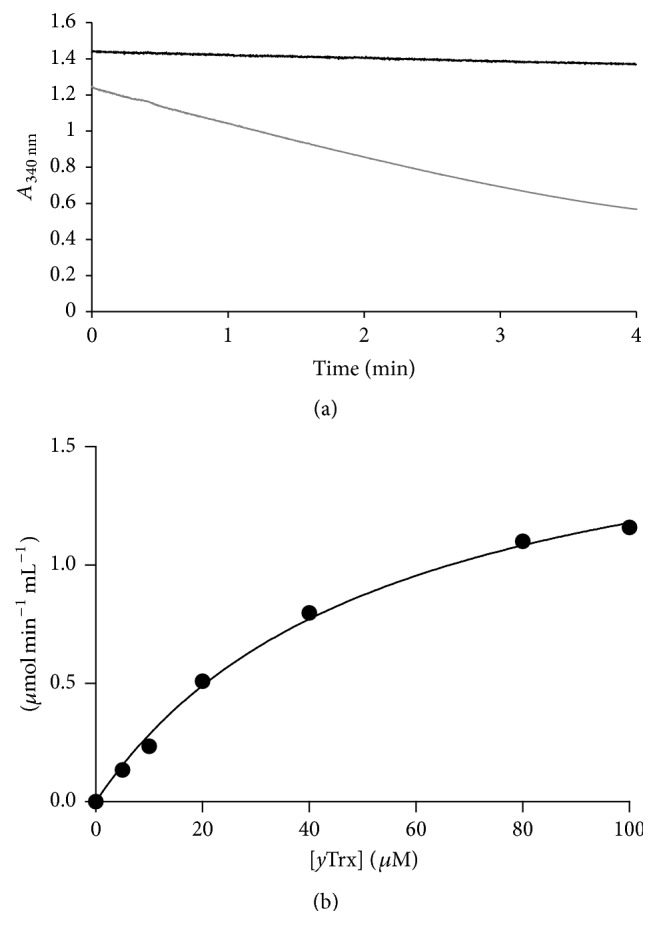
Bcp1 peroxidase activity is dependent on* y*TrxR/*y*Trx. The activity was measured by the consumption of NADPH via a decrease in absorbance at 340 nm. (a) Time course of NADPH oxidation: the peroxidase activity of Bcp1 (3.5 *μ*M) was measured at 30°C in presence of* y*TrxR (0.1 *μ*M) and* y*Trx (0.1 mM). The reaction was started by adding 0.2 mM H_2_O_2_. (b) Michaelis-Menten plot of Bcp1 using* y*Trx as the substrate.

It was previously described that Bcp1 is coupled with an unusual redox system composed of* Ss*TrxR with* Ss*PDO for recycling [4]. We monitored the Bcp1 peroxidase reaction using the yeast Trx system (*y*TrxR/*y*Trx) and NADPH as an electron source to verify that Bcp1 activity was supported by eukaryotic disulfide reductase system. This system was successfully used to measure the peroxidase activity of* h*Prx1 and* h*Prx2 [18, 24]. Therefore, we expressed and purified* y*TrxR and* y*Trx from* E. coli* and determined their reducing activities, as reported in the MM (data not shown). Initially, Bcp1 peroxidase activity was determined by measuring the consumption of NADPH, which was revealed by a decrease in absorbance at 340 nm (Figure 3(a)); then, different concentrations of* y*Trx were used to determine the kinetic parameters (Figure 3(b)). Our results showed that Bcp1 had a *K*_*m*_ value of 54.9 *μ*M using this heterologous system.

### 3.3. Generation of a Pure TAT-Bcp1 Fusion Protein

Previous studies showed that when the HIV Transactivating Transduction (TAT) domain, a short basic polypeptide of 11 amino acids residues, was fused with a target protein it confers the ability to penetrate across the lipid bilayer of the cells due to the strong binding of the positively charged TAT domain to the negative charges on the cell surface [17].

We generated the TAT-Bcp1 fusion protein by cloning* bcp1* into pTAT2.2 vector to allow Bcp1 internalization into the cell. The recombinant plasmid, pTAT2.2-Bcp1 (Figure S1A), was used to produce a soluble fusion protein with the TAT domain at the N-terminus and a His tag at the C-terminus. TAT-Bcp1 was purified by heat-treatment followed by IMAC chromatography using the conditions described for Bcp1 [4]. The SDS-PAGE analysis (Figure S1B) revealed a single band of ~19 kDa, which agreed with the predicted molecular weight of 20.229 kDa.

The purified enzyme was functionally characterized. The DTT assay was performed and the results revealed that, similar to Bcp1, TAT-Bcp1 was able to reduce all of the H_2_O_2_ under the same pH and temperature conditions (data not shown).

### 3.4. TAT-Bcp1 Internalization into H9c2 Cells

**Figure 4:**
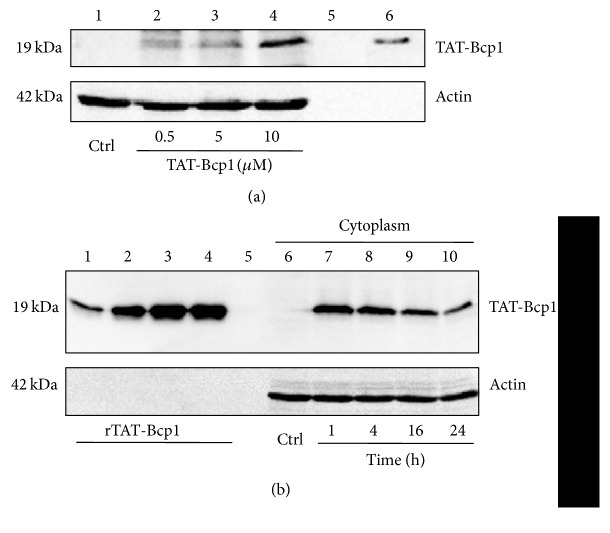
Transduction of TAT-Bcp1 in H9c2 cells. A western blot analysis of the cytoplasmic extracts was performed to detect TAT-Bcp1 in the H9c2 cells; the cytoplasmic proteins were analysed with a penta-His HRP-conjugated mAb. Actin was used as the cytoplasmic marker (lower panels). (a) H9c2 cells were cultured in growth medium* (lane 1)* and incubated with 0.5 *μ*M* (lane 2)*, 5 *μ*M* (lane 3)*, and 10 *μ*M* (lane 4)* TAT-Bcp1 for 1 h. Purified TAT-Bcp1 was loaded as reference (*lane 6*). (b) H9c2 cells were cultured in growth medium* (lane 6)* and incubated with TAT-Bcp1 (10 *μ*M) for 1 h* (lane 7)*, 4 h* (lane 8)*, 16 h* (lane 9),* and 24 h* (lane 10)*. Increasing amounts of purified TAT-Bcp1 were loaded to construct a calibration curve to determine the intracellular TAT-Bcp1 levels: 25 ng* (lane 1)*, 50 ng* (lane 2)*, 75 ng* (lane 3)*, and 100 ng* (lane 4)*.

It is widely reported that H_2_O_2_ contributes to the initiation and the progression of cardiovascular diseases, including atherosclerosis [26]. We chose H9c2 rat cardiomyoblasts, nonmalignant cardiac-like cells that are commonly used to study the molecular response to oxidative damage [27], as a model system to evaluate the effects of TAT-Bcp1 on cells undergoing oxidative stress. First, we established the optimal conditions for TAT-Bcp1 internalization. Therefore, we cultured the H9c2 cells and incubated them with different amounts (0–10 *μ*M) of the fusion protein for 1 h. As shown in Figure 4(a), the western blot analysis of the cytoplasmic extracts showed that 10 *μ*M TAT-Bcp1 was the optimal concentration for internalization into H9c2 cells. Afterwards, TAT-Bcp1 internalization was analysed at 1 h, 4 h, 16 h, and 24 h (Figure 4(b)). The fusion protein was detected in the cells after 1 h (lane 7, upper panel), whereas 16%, 49%, and 69% decreases were observed after 4 h, 16 h, and 24 h, respectively (lanes 8–10, upper panel). This effect could be related to the common protein turnover mechanism because a constant amount of TAT-Bcp1 was present in the supernatants at the different times (Figure S2, lanes 6–9 (see Supplementary Material available online at http://dx.doi.org/10.1155/2016/7424870)). No band was detected when buffer alone was diluted into the culture medium (Figure 4(a), lane 1, and Figure 4(b), lane 6, upper panels; Figure S2, lane 5). These data indicate that TAT-Bcp1 is optimally delivered into the cells at 10 *μ*M and is stable within 4 h after administration.

### 3.5. The Effect of TAT-Bcp1 on Cell Viability

**Figure 5:**
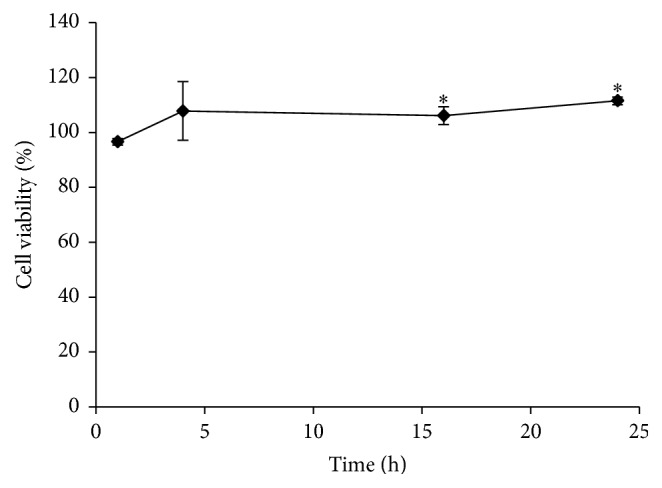
Effect of TAT-Bcp1 on H9c2 cell viability. Cell viability was determined using the MTT assay. The cells were cultured in normal growth medium and then treated with TAT-Bcp1 (10 *μ*M) for 1 h, 4 h, 16 h, and 24 h. Cell viability was evaluated by measuring the *A*_570 nm_; the data are expressed as the mean percentages ± SD. compared to the control. ^*∗*^ *p* < 0.001; the results were derived from four replicates of a representative experiment.

The MTT assay was performed to evaluate whether TAT-Bcp1 had any cytotoxic effect on the H9c2 cells. The cells were cultured in growth medium and treated with 10 *μ*M TAT-Bcp1 for 1 h, 4 h, 16 h, and 24 h (Figure 5). After a 1 h incubation, cell growth was 96.6 ± 1.2%, whereas after 16 h and 24 h, the cell survival was slightly but significantly higher than 100% (106.1 ± 3.2 and 111.5 ± 1.4%, resp.; *p* value < 0.01), showing that TAT-Bcp1 was not cytotoxic to H9c2 cells. The incubation was also extended to 72 h (Figure S3), and the results showed that 95% and 91% of the cells survived at 48 h and 72 h, respectively, confirming the data described above.

### 3.6. TAT-Bcp1 Affects the H_2_O_2_ Concentrations in H9c2 Cells under Physiological or Stressed Conditions

The endogenous H_2_O_2_ content was measured in lysates of H9c2 cells that had been treated with increasing concentrations of TAT-Bcp1 and were or were not subjected to oxidative stress to establish the effect of TAT-Bcp1 on the H_2_O_2_ content in H9c2 cells.

**Figure 6:**
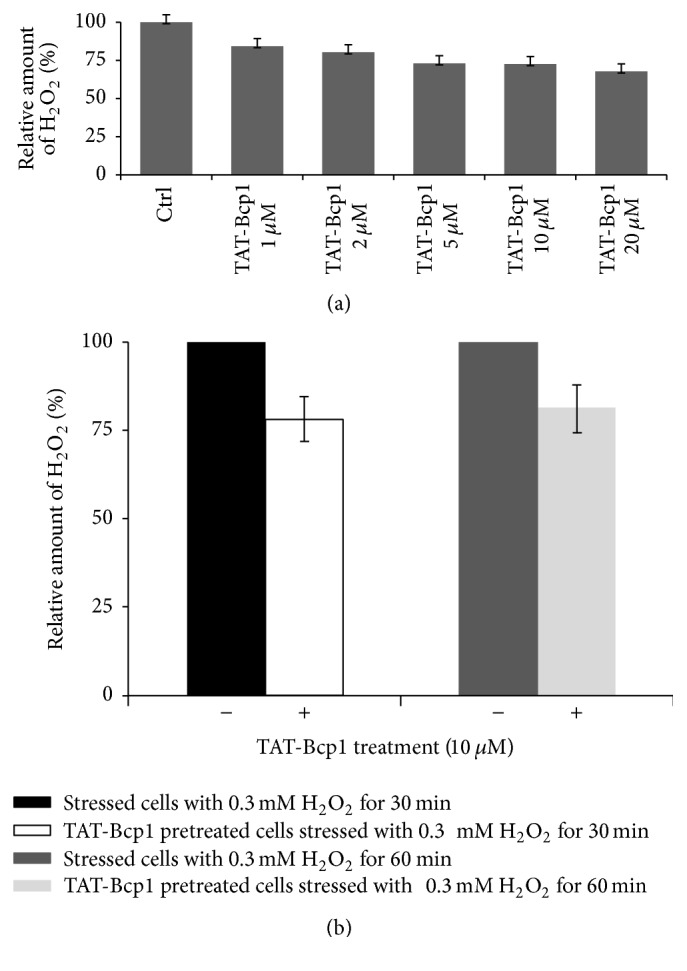
Effect of TAT-Bcp1 on H_2_O_2_ levels in H9c2 cells. (a) Dose-dependent effect of TAT-Bcp1 (1–20 *μ*M) on the H_2_O_2_ content in H9c2 cells grown under physiological conditions. (b) Effect of the TAT-Bcp1 pretreatment on unstressed and H_2_O_2_-stressed cells. Unstressed cells (dark bars); H_2_O_2_-stressed cells (light bars).

In the first case, the H_2_O_2_ levels in H9c2 cell lysates were determined after incubation with increasing amounts (1 to 20 *μ*M) of TAT-Bcp1 for 1 h at 37°C. The H_2_O_2_ levels in the lysates were determined using the Fluorescent Peroxide Sensor (see Section 2.7). As shown in Figure 6(a), a decrease of H_2_O_2_ content was detected in a TAT-Bcp1 dose-dependent manner, indicating that the protein was able to cooperate with the endogenous reducing systems for H_2_O_2_ removal.

In the latter case, we investigated the antioxidant activity of TAT-Bcp1 by measuring the H_2_O_2_ levels in cells subjected to oxidative stress. As shown in Figure 6(b), the H_2_O_2_ levels in the TAT-Bcp1 pretreated, stressed cells (see methods) were significantly reduced compared to the untreated samples. Taken together, these results indicate that TAT-Bcp1 reduces the H_2_O_2_ content in H9c2 cells under both physiological and oxidative stress conditions.

### 3.7. TAT-Bcp1 Protects H9c2 Cardiomyoblasts from H_2_O_2_-Induced Cell Apoptosis

**Figure 7:**
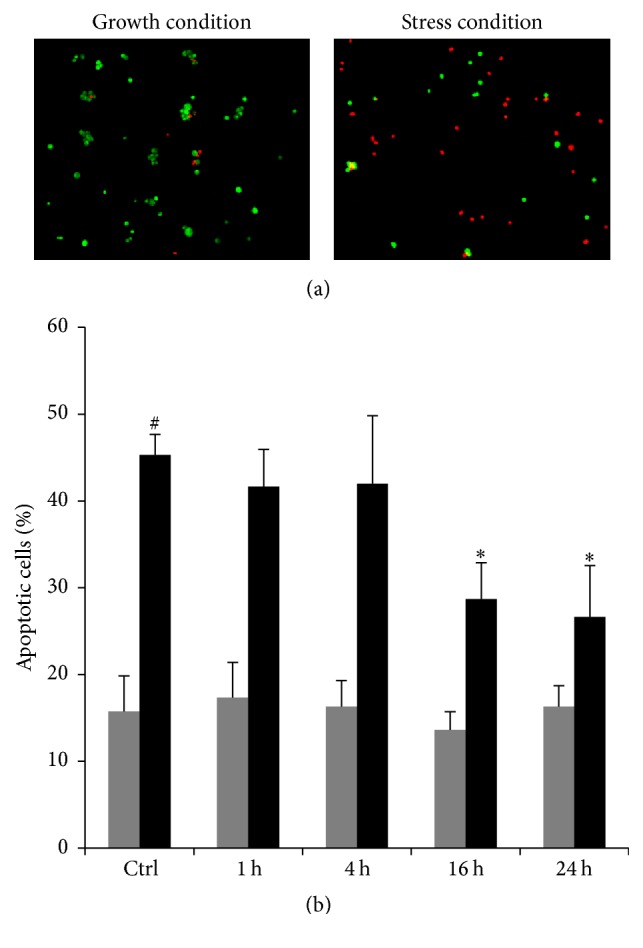
Effect of TAT-Bcp1 on cell apoptosis. Cell apoptosis was evaluated using EB and AO staining. H9c2 cells were treated with TAT-Bcp1 (10 *μ*M) for different times (1 h, 4 h, 16 h, and 24 h) and then subjected to oxidative stress with 0.3 mM H_2_O_2_ for 1 h. (a) Representative images of the control cells (untreated with TAT-Bcp1) under growth and stress conditions showing both apoptotic (red) and live (green) cells. (b) The graph shows the mean percentages ± SD. of apoptotic cells. The grey and black bars represent the unstressed and stressed cells, respectively. ^#^ *p* ≪ 0.01 versus the Ctrl growth condition; ^*∗*^ *p* ≪ 0.001 versus the Ctrl stress condition.

The H9c2 cells were pretreated with 10 *μ*M TAT-Bcp1 for different incubation times (1–24 h) and then exposed to 0.3 mM H_2_O_2_ for 1 h to study the effect of TAT-Bcp1 on H_2_O_2_-induced apoptosis. Cell apoptosis was analysed by EB and AO staining (Figure 7(a)).

A significant increase in the number of apoptotic cells was observed following the induction of oxidative stress; the percentage of H_2_O_2_-treated apoptotic cells was approximately 3-fold higher (45.3 ± 2.3%) (Figure 7(b), Ctrl black bar) than the unstressed cells (15.7 ± 4.1%) (Figure 7(b), Ctrl grey bar). Pretreatment with TAT-Bcp1 produced a protective effect against H_2_O_2_-induced cell apoptosis in a time-dependent manner. Indeed, after 1 h and 4 h incubation with TAT-Bcp1, the percentage of apoptotic cells was 41.6 ± 4.3% and 42 ± 7,8%, respectively, similar to that observed under stress conditions. In contrast, at 16 h and 24 h, the percentage of apoptotic cells was significantly reduced 1.6-fold (28.7 ± 4.2% and 26.6 ± 5.9%, resp.). Furthermore, the administration of TAT-Bcp1 to the unstressed cells did not induce any increase in cell apoptosis (Figure 7(b), grey bars), consistent with the results obtained from the MTT assay.

**Figure 8:**
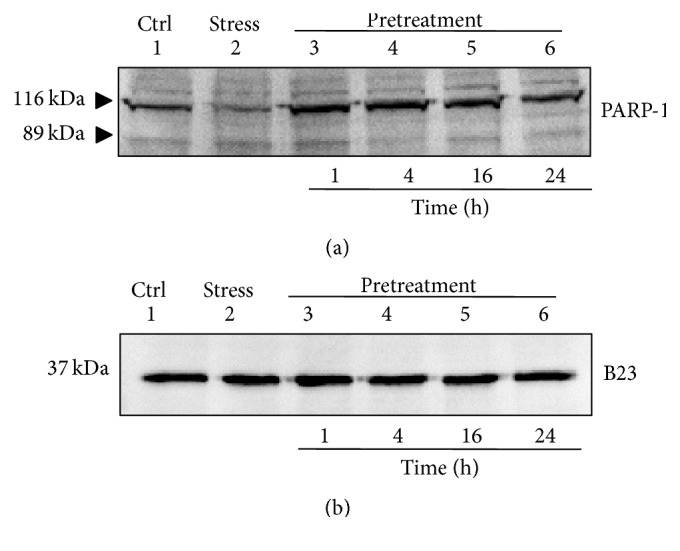
PARP-1 cleavage analysis. A western blot analysis of PARP-1 was performed using nuclear extracts. H9c2 cells were cultured under growth* (lane 1)* and stress conditions with 0.3 mM H_2_O_2 _for 3 h* (lane 2)* or pretreated with TAT-Bcp1 (10 *μ*M) for different times, 1 h* (lane 3)*, 4 h* (lane 4)*, 16 h* (lane 5)*, and 24 h* (lane 6)*, before oxidative stress was induced (a). B23 was used as the nuclear marker (b). Both the 116 kDa full-length fragment and 89 kDa cleaved fragment are shown.

PARP-1 cleavage was examined by a western blot analysis of nuclear extracts to confirm the effect of TAT-Bcp1 on H_2_O_2_-induced cell apoptosis (Figure 8). Typical PARP-1 cleavage was detected under the stress conditions induced by incubating the cells with 0.3 mM H_2_O_2_ for 3 h (Figure 8, lane 2), whereas pretreatment with 10 *μ*M TAT-Bcp1 inhibited PARP-1 cleavage, showing a pattern that was similar to the control (Figure 8, lanes 3–6). In particular, under stress conditions, the level of the full-length fragment showed a 25% decrease compared to the control, whereas, after the TAT-Bcp1 pretreatment, the level of the full-length fragment level was 94% after 1 h and 100% after 4, 16, and 24 h, showing a cellular response that was comparable among the different pretreatment times.

In accord with the data from the EB and AO assays, the PARP-1 cleavage analysis confirms the antiapoptotic effect of TAT-Bcp1 in H_2_O_2_-stressed cells.

## 4. Discussion

Prxs have recently received more attention because they are very efficient at removing low concentrations of H_2_O_2_ due to both their high abundance and low *K*_*m*_ (~20 *μ*M) [28, 29]. Prxs, which are usually expressed in different isoforms, are evolutionarily conserved from the Archaea, such as* S. solfataricus*, in which four enzymes have been extensively characterized, to man, in which six isoenzymes with different roles and subcellular localizations have been identified. The ubiquity of Prxs and their homology conserved among the different phylogenetic domains show the important roles of these enzymes in addition to their peroxidase activities [30]. Although they retained the Trx fold that characterizes this family and peroxidase activity [2], these enzymes have implemented their functions by inserting a sensitivity motif in their structure to achieve fine tuning of the peroxide levels and play a regulatory role in cells. Growing evidence suggests both the unquestionable involvement of Prxs in the oxidative stress response and their role in regulating the intracellular H_2_O_2_ levels, which are responsible for cell proliferation, angiogenesis, and apoptosis [31–33]. Furthermore, it was shown that the sensitivity motif that promotes the overoxidation of Prx evolved independently in eukaryotic and prokaryotic species [34].

Our studies have focused on the antioxidant activity of a robust Prx to develop new antioxidant drugs; we chose prokaryotic Prx, Bcp1, from* S. solfataricus* that lacks the sensitivity motif and has a higher robustness compared to its eukaryotic counterparts. Starting from these differences, we first assessed whether Bcp1 exhibited peroxidase activity under conditions that are typical of mammalian cells and in the presence of the eukaryotic recycling system yTrxR/yTrx. Our results showed that the enzyme can function at 37°C and pH 7.0 and is still stable after 16 h incubation under these conditions, highlighting the potential use of this enzyme in biotechnological applications [35]. In contrast to eukaryotic and bacterial Prxs, Bcp1 has a peculiar recycling system composed of TrxR/PDO [13] instead of the TrxR/Trx reducing system that is generally used by eukaryotes. Consequently, it was not obvious whether Bcp1 could interact with yTrx in the hybrid regenerating system. Our results not only showed that Bcp1 was effective at reducing the H_2_O_2_ levels in the presence of the* y*TrxR/*y*Trx system but also showed that its affinity with* y*Trx was comparable to that reported for human Prxs [18], suggesting a possible activity of Bcp1 in eukaryotic cells. Based on the* in vitro* characterization, we analysed the effect of Bcp1 on H_2_O_2_-induced apoptosis into H9c2 cells, with the aim of analysing the possible use of the archaeal Prx as an antioxidant.

We designed the fusion protein TAT-Bcp1 to mediate its internalization into H9c2 cells and to analyse the effect of Bcp1 on the cell stress response. TAT-Bcp1 was effectively detected in the cells at 1 h after administration and gradually decreased to 70% after 24 h, in agreement with the protein turnover rates. Furthermore, the protein did not induce any cytotoxic effects, but it could have a cytoprotective effect, as shown by the slight but significant increase in cell viability at 16 h and 24 h. The results obtained in this study indicate that TAT-Bcp1 not only efficiently decreased the H_2_O_2_ levels in H9c2 cells under both physiological and stressed conditions, showing that the enzyme can use the endogenous recycling system, but was also able to reduce apoptosis, as shown in the EB/AO staining assay in Figure 7 and in the PARP-1 cleavage analysis in Figure 8. Together, these results suggest that the antioxidant activity of TAT-Bcp1 could play a role in protecting cells from apoptosis.

## 5. Conclusions

This study is the first report of an archaeal enzyme delivered into mammalian cultured cells that is able to protect cells from oxidative stress by reducing both the peroxide levels inside the cells and the resulting apoptosis. The intrinsic stability of Bcp1 and its modification, which provides the ability to penetrate cells, makes TAT-Bcp1 a good candidate to study the effectiveness of scavenging ROS in the cell. Growing evidence is highlighting the roles of Prxs in different diseases, ranging from diabetes to neurological disorders to cardiovascular diseases; therefore, this work aims to provide new insights into the possibility of using alternative sources of antioxidant enzymes, such as those from thermophilic microorganisms.

## Supplementary Material

Figure S1: Cloning and expression of the TAT-Bcp1 fusion protein. (A) pTAT2.2-Bcp1 map. The T7 promoter region and TAT sequence are indicated by the black lines upstream of the bcp1coding region (grey). The His-tag is indicated by a dashed line downstream of bcp1. NdeI and XhoI are the restriction sites used for cloning. (B) SDS-PAGE analysis of the different purification steps for TAT-Bcp1. Lane 1: molecular weight markers (Protein Marker VI-Applichem); lane 2: *E. coli* BL21-CodonPlus (DE3)-RIL cellular extract; lane 3: *E. coli* BL21-CodonPlus (DE3)-RIL cellular extract induced with IPTG; lane 4: heat-treated cellular extracts; lane 5: IMAC chromatography.
Figure S2: Detection of extracellular TAT-Bcp1. A western blot analysis was performed to detect TAT-Bcp1 in the medium of cultured H9c2 cells; the same amounts of conditioned medium were analysed with the penta-His HRP conjugated mAb. H9c2 cells were cultured in growth medium (lane 5) and incubated with TAT-Bcp1 (10 µM) for 1 h (lane 6), 4 h (lane 7), 16 h (lane 8) and 24 h (lane 9). Decreasing amounts of purified TAT-Bcp1 were loaded to construct a calibration curve: 100 ng (lane 1), 75 ng (lane 2), 50 ng (lane 3), and 25 ng (lane 4).
Figure S3: Effect of TAT-Bcp1 on H9c2 cell viability at different incubation times. Cell viability was determined using the MTT assay. The cells were cultured in normal growth medium and then treated with TAT-Bcp1 (10 µM) for 24 h, 48 h and 72 h. Cell viability was evaluated by measuring the A570nm; the data are expressed as the mean percentages ± S.D. compared to the control. ∗*p* < 0.001; the results were derived from four replicates of a representative experiment.
