# Supplementary material for: Discovering Antioxidant Molecules in the Archaea Domain: Peroxiredoxin Bcp1 from Sulfolobus solfataricus Protects H9c2 Cardiomyoblasts from Oxidative Stress
Source: Archaea. 2016 Sep 26;2016:7424870. doi: 10.1155/2016/7424870 (PMC5056240; doi:10.1155/2016/7424870)
Supplement: Supplementary file 1 — Figure S1: Cloning and expression of the TAT-Bcp1 fusion protein. (A) pTAT2.2-Bcp1 map. The T7 promoter region and TAT sequence are indicated by the black lines upstream of the bcp1coding region (grey). The His-tag is indicated by a dashed line downstream of bcp1. NdeI and XhoI are the restriction sites used for cloning. (B) SDS-PAGE analysis of the different purification steps for TAT-Bcp1. Lane 1: molecular weight markers (Protein Marker VI-Applichem); lane 2: E. coli BL21-CodonPlus (DE3)-RIL cellular extract; lane 3: E. coli BL21-CodonPlus (DE3)-RIL cellular extract induced with IPTG; lane 4: heat-treated cellular extracts; lane 5: IMAC chromatography. Figure S2: Detection of extracellular TAT-Bcp1. A western blot analysis was performed to detect TAT-Bcp1 in the medium of cultured H9c2 cells; the same amounts of conditioned medium were analysed with the penta-His HRP conjugated mAb. H9c2 cells were cultured in growth medium (lane 5) and incubated with TAT-Bcp1 (10 µM) for 1 h (lane 6), 4 h (lane 7), 16 h (lane 8) and 24 h (lane 9). Decreasing amounts of purified TAT-Bcp1 were loaded to construct a calibration curve: 100 ng (lane 1), 75 ng (lane 2), 50 ng (lane 3), and 25 ng (lane 4). Figure S3: Effect of TAT-Bcp1 on H9c2 cell viability at different incubation times. Cell viability was determined using the MTT assay. The cells were cultured in normal growth medium and then treated with TAT-Bcp1 (10 µM) for 24 h, 48 h and 72 h. Cell viability was evaluated by measuring the A570nm; the data are expressed as the mean percentages ± S.D. compared to the control. ∗p < 0.001; the results were derived from four replicates of a representative experiment. [file 7424870.f1.pdf]

A

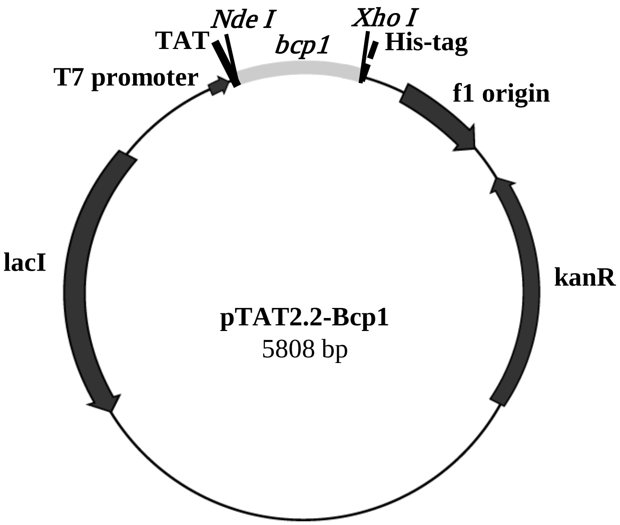

B

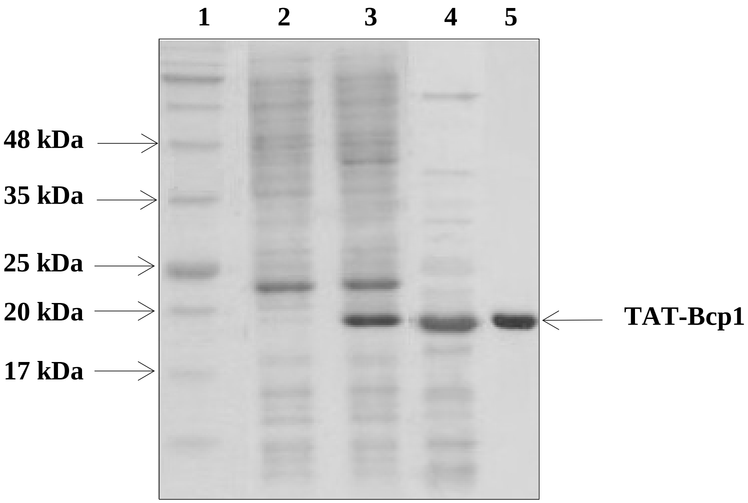

Figure S1: Cloning and expression of the TAT-Bcp1 fusion protein. **(**A) pTAT2.2-Bcp1 map. The T7 promoter region and TAT sequence are indicated by the black lines upstream of the *bcp1*coding region (grey). The His-tag is indicated by a dashed line downstream of *bcp1*. *NdeI* and *XhoI* are the restriction sites used for cloning. (B) SDS-PAGE analysis of the different purification steps for TAT-Bcp1. *Lane 1*: molecular weight markers (Protein Marker VI-Applichem); *lane 2*: *E. coli* BL21-CodonPlus (DE3)-RIL cellular extract; *lane 3*: *E. coli* BL21-CodonPlus (DE3)-RIL cellular extract induced with IPTG; *lane 4*: heat-treated cellular extracts; *lane 5*: IMAC chromatography

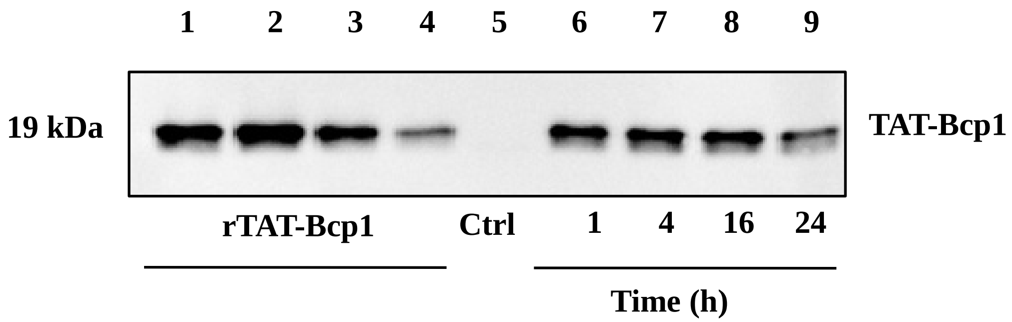

Figure S2: Detection of extracellular TAT-Bcp1. A western blot analysis was performed to detect TAT-Bcp1 in the medium of cultured H9c2 cells; the same amounts of conditioned medium were analysed with the penta-His HRP conjugated mAb. H9c2 cells were cultured in growth medium (*lane 5*) and incubated with TAT-Bcp1 (10 µM) for 1 h (*lane 6*), 4 h (*lane 7*), 16 h (*lane 8*) and 24 h (*lane 9*). Decreasing amounts of purified TAT-Bcp1 were loaded to construct a calibration curve: 100 ng (*lane 1*), 75 ng (*lane 2*), 50 ng (*lane 3*), and 25 ng (*lane 4*).

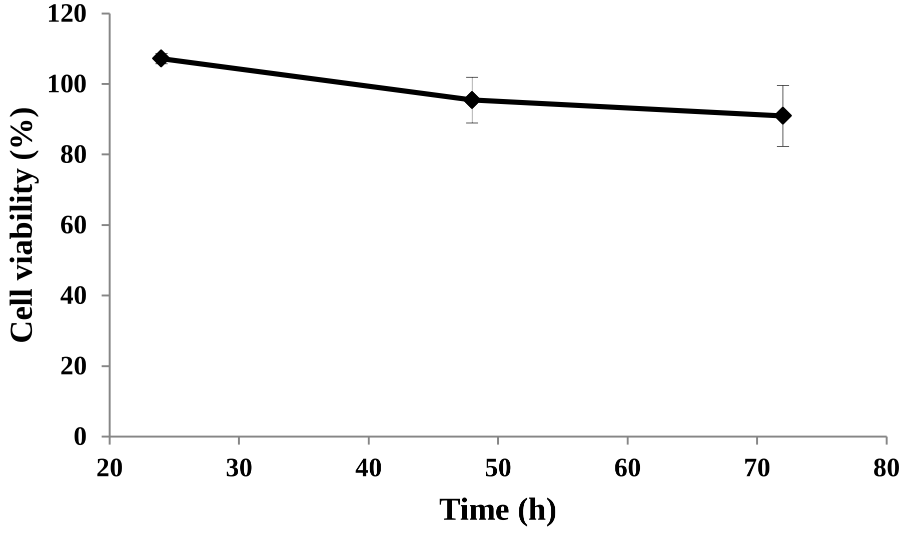

Figure S3: Effect of TAT-Bcp1 on H9c2 cell viability at different incubation times. Cell viability was determined using the MTT assay. The cells were cultured in normal growth medium and then treated with TAT-Bcp1 (10 µM) for 24 h, 48 h and 72 h. Cell viability was evaluated by measuring the A_{570nm}; the data are expressed as the mean percentages ± S.D. compared to the control. * $p < 0.001$; the results were derived from four replicates of a representative experiment.
